# B7-1 drives TGF-β stimulated pancreatic carcinoma cell migration and expression of EMT target genes

**DOI:** 10.1371/journal.pone.0222083

**Published:** 2019-09-04

**Authors:** Jeong-Han Kang, Mi-Yeon Jung, Edward B. Leof

**Affiliations:** Departments of Medicine and Biochemistry & Molecular Biology, Division of Pulmonary and Critical Care Medicine, Thoracic Disease Research Unit, Mayo Clinic College of Medicine, Rochester, Minnesota, United States of America; University of Nebraska Medical Center, UNITED STATES

## Abstract

B7-1 proteins are routinely expressed on the surface of antigen presenting cells (APC) and within the innate immune system. They function to establish a biologically optimal and dynamic balance between immune activation and inhibition or self-tolerance. Interactions between B7-1 and its receptors, which include CD28, CTLA4 and PD-L1, contribute to both stimulatory as well as inhibitory or homeostatic regulation. In the current study, we investigated whether the tumor-promoting actions of transforming growth factor beta (TGF-β) disrupted this equilibrium in pancreatic cancer to promote malignant progression and an enhanced means to evade immune detection. The data show that B7-1 is (i) upregulated following treatment of pancreatic carcinoma cells with TGF-β; (ii) induced by TGF-β via both Smad2/3-dependent and independent pathways; (iii) required for pancreatic tumor cell in vitro migration/invasion; and (iv) necessary for TGF-β regulated epithelial-mesenchymal transition (EMT) through induction of Snail family members. Results from the proposed studies provide valuable insights into mechanisms whereby TGF-β regulates both the innate immune response and intrinsic properties of pancreatic tumor growth.

## Introduction

The American Cancer Society has reported that approximately 44,330 people will die from pancreatic cancer in Cancer Facts & Figures 2018. Although this reflects the 4th leading cause of cancer-associated deaths in the United States, unfortunately, recent advances in diagnostics, staging, and therapy have not resulted in significant improvements in long-term survival. A major component impacting pancreatic cancer’s mortality is its ability to maintain an immunosuppressive microenvironment characterized by poor T cell penetration [1]. Furthermore, the known ability of pancreatic cancer antigens to generate relatively weak immune responses coupled with the expression of multidrug-resistant genes and growth factors such as transforming growth factor-β (TGF-β), tumor-necrosis factor-α (TNFα), and vascular endothelial growth factor (VEGF) help provide optimal conditions to promote tumor growth [2–5]. While these proliferative signals are counter-acted by the host’s adaptive immune response [6], immunoediting can give rise to tumor variants capable of escaping immune surveillance [7]. Additional cancer promoting activities reflect the induction or recruitment of immunomodulatory cells such as regulatory T (Treg) cells, type 2 macrophages, and myeloid-derived suppressor cells as well as immunosuppressive cytokines including IL-10 and TGF-β [8–11]. The manner by which these various immune subversion strategies function are varied and include, but are not limited to, down-regulation of MHC class I or co-stimulatory molecules on antigen presenting cells (APC) or enhancement of co-inhibitory/checkpoint molecules [8, 10].

As immune checkpoint molecules play an essential role in tumor progression [12], blocking their action provides a hopeful approach for triggering anti-tumor immunity [13]. For instance, antibodies targeting two B7/CD28 family checkpoint proteins, CTLA-4 and PD-1, have recently been approved for the treatment of advanced melanoma [14–16] and B7-1 expression has been reported to be increased in pancreatic cancer [17, 18]. SinceTGF-β is a known negative regulator of the immune response [17–19], we wished to determine if B7 ligands were coupled to the tumor-promoting activity of TGF-β.

TGF-β is a 25 kDa homodimeric peptide involved in numerous homeostatic as well as pathological processess [20, 21]. Following binding and activation of an heteromeric receptor complex [22, 23], downstream signlaing is primarily mediated by the Smad proteins, Smad2 and Smad3. These 42–60 kDa proteins become phosphorylated by the type I TGF-β receptor, form hetero-oligomeric complexes with Smad4, and translocate to the nucleus where they impact gene transcription [24, 25]. In addition to the canonical Smad pathway, TGF-β activity is also regulated via non-canonical Smad-independent mechanisms [21, 26–28]. As such, in the current study we investigated, *first*, if TGF-β induced the expression of the negative immune modulators B7-1 and/or B7-2 in pancreatic cancer, and if so, *second*, whether checkpoint protein expression was mediated via Smad and/or non-Smad signaling and necessary for aspects of TGF-β’s protumorigenic actions.

## Materials and methods

### Cell culture

Human Pancreatic (PANC-1 and PANC04.03) and murine mKPC cells were obtained, respectively, from the American Type Culture Collection (ATCC) and graciously provided by Dr. David Tuveson (Cold Spring Harbor Laboratory). All cells were cultured at 37°C in 5% CO_2_ in Dulbecco's modified Eagle's medium (DMEM) (high glucose) (Gibco, Grand Island, NY) containing 10% fetal bovine serum (FBS). Unless stated otherwise, 2.5 X 10^5^ cells were seeded into 6 well plates and following overnight incubation at 37°C placed into 0.1% FBS/DMEM for 18 hr. The medium was then replaced with fresh 0.1% FBS/DMEM containing the indicated supplements and processed as indicated.

### Protein knockdown by siRNA

Cells were transiently transfected with 60 nmol/L B7-1 siRNAs (sc-39699), Smad2/3 siRNAs (sc-37238 and sc-37239) or non-targeting siRNAs (sc-37007) as control siRNA according to the manufacturer's instructions (Santa Cruz Inc, CA, USA). Briefly, 2.5 × 10^5^ cells were incubated in DMEM with 10%FBS and then transfected with the indicated siRNAs using Lipofectamine (Invitrogen, Carlsbad, CA, USA) according to the manufacturer's instructions. The next day, following 24 hr recovery in complete medium the effect of knockdown on target gene or protein expression was determined, respectively, by RT-qPCR or Western blotting.

### RT-qPCR analysis

Total RNA was isolated from 5 X 10^6^ cells using a RNeasy Plus Mini kit (Qiagen, Valencia, CA, USA). 500 ng RNA was used for cDNA synthesis using random hexamers and SuperScript III reverse transcriptase (Invitrogen, Carlsbad, CA, USA) in a 30 μl reaction. Five microliter of the resulting cDNA was used for real-time PCR using the TaqMan gene expression assay for B7-1 (Hs01045161_m1), TWIST1 (Hs01675818_s1), TWIST2 (Hs02379973_s1), SNAIL1 (Hs00195591_m1), SNAIL2(Hs00161904_m1), ZEB1 (Hs01566408_m1), ZEB2 (Hs00207691_m1), COL1A1(Hs00164004_m1), FN1(Hs01549976_m1) and ACTB (Hs01060665_g1) according to the manufacturer’s instructions (Applied Biosystems, Pleasanton CA,USA). All experiments were performed in triplicate and relative expression levels were determined by the 2^-ΔΔCt^ method [29].

### Western blotting

Following treatment with the indicated reagents, cellular lysates were prepared on ice with RIPA buffer (50 mM Tris [pH 7.4], 1% Triton X100, 0.25% Sodium deoxycholate, 150 mM NaCl, 1 mM EDTA [pH 8] and 10 mM NaF) containing cOmplete Protease Inhibitor (Roche, Indianapolis, IN, USA). Insoluble material was removed by centrifugation (18,000 g for 10 min) and 15–20 μg protein was processed for Western analysis following 10% SDS-PAGE. Detection was by enhanced chemiluminescence using antibodies to B7-1 (Abcam, Ab86473 and Santa Cruz, Sc-376012), B7-2 (Santa Cruz, SC28347), N-cadherin (BD, 616920), Vimentin (Cell signaling, 3932s), Snail (Cell signaling, 3895s) and GAPDH (EMD Millipore, MAB374). pSmad2 and pSmad3 antibodies were generated and used as previously described [30].

### Scratch assay

For scratch assays, 3 X 10^5^ cells in 10% FBS/DMEM were seeded into 6 well plates. The next day, following disruption of the monolayer with a sterile 200 μl pipet tip the cultures were washed and incubated ± TGF-β for 24 hours at 37°C in 0.1% FBS/DMEM. Images were obtained with an EVOS XL Core Imaging System at 0 and 24 hr.

### Migration assay

For migration assays, 1 X 10^5^ cells in 0.1% FBS/DME were seeded in the upper chamber of 8 μm pore size transwell plates (Costar, Cambridge, MA). Following 24 hr incubation at 37°C, any remaining cells in the upper chamber were removed with a cotton swab, and cells which migrated to the lower chamber were fixed and stained using a Differential Quik Stain Kit according to the manufacturer's instructions (Polysciences, Inc., PA, USA). Each experiment was performed in duplicate and cell counting was done in 3 randomly selected fields.

### Immunohistochemistry

Normal human and pancreatic cancer frozen sections were obtained from US Biomax (Rockville, MD, USA). The Mayo Clinic Institutional Review Board (IRB) acknowledges that based on review of the study it does not require IRB review as the human tissue samples were confirmed to be de-identified. Slides were permeabilized with 0.1% saponin/PBS for 10 min at room temperature and then incubated overnight at 4°C with primary antibodies to B7-1 (1:200, Abcam [Ab86473]) or CK-19 (1:200 Abcam [Ab52625]) in 1% BSA, 0.1% saponin, PBS. Following incubation in the dark for 1 hr with secondary antibodies (Alexa 488 [1:200, green] or Alexa 594 [1:200 red]; Invitrogen, Carlsbad, CA, USA), slides were washed with PBS and images obtained using confocal microscopy (Carl Zeiss, Thornwood, NY, USA). Co-localization of B7-1 and CK-19 was quantitated using ImageJ software (National Institutes of Health, Bethesda, MD, USA)

### Flow cytometry

2.5 X 10^5^ cells were seeded into 6 well plates and following overnight incubation the medium was replaced with fresh 0.1% FBS/DMEM at 37°C for 18 hr. Cultures were then placed in fresh 0.1% FBS/DMEM for 24 hr ± TGF-β, trypsinized, washed, and then 1 X 10^5^ cells stained with B7-1-Alexa 596 (BD Pharmingen San Jose, CA) for 30 min at room temperature. After MACS buffer (Miltenyi Biotec, San Diego, CA) washing and 2% paraformaldehyde fixation, FACScan flow cytometer was performed by gating 1 X 10^4^ living cells.

### Statistical analysis

All data are presented as mean ± SEM. Two-tailed Student’s *t*-test or ANOVA was used to evaluate statistical significance using GraphPad Prism 5 software (GraphPad Software, Inc). *P* < 0.05 was considered statistically significant.

## Results

### B7-1 (CD80) is highly expressed in human pancreatic cancer and regulated by TGF-β in human pancreatic cancer cells

**Fig 1:**
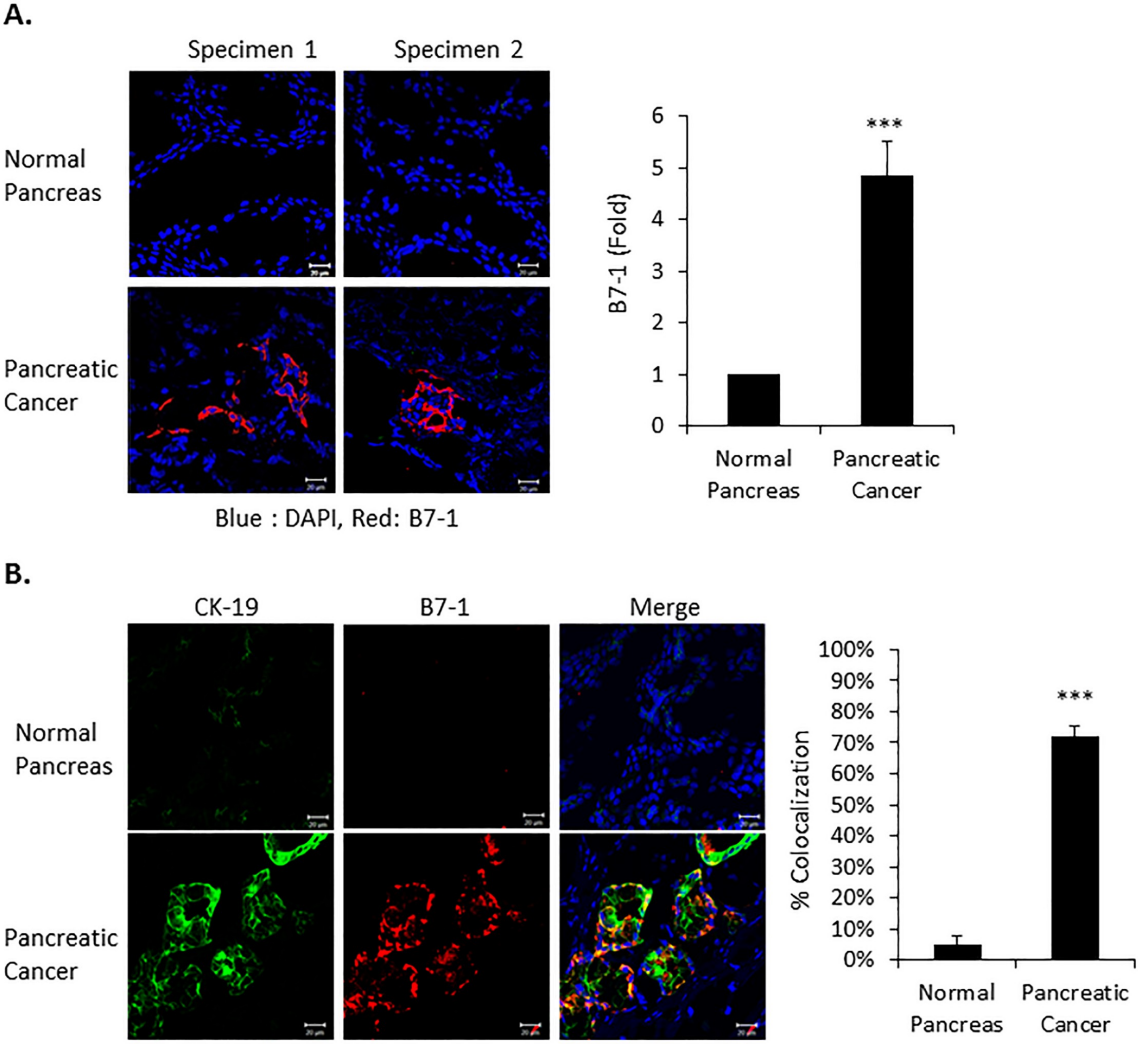
B7-1 expression in human pancreatic cancer. (A) Frozen normal (n = 2) and human pancreatic tumor tissue (n = 2) was obtained from US Biomax (Rockville, MD, USA). Immunofluorescence confocal microscopy was performed on 3 slides from each of the two Normal and Pancreatic Cancer tissue specimens for B7-1 (red) and nuclear DAPI (blue). Expression of B7-1 is quantitated in the right panel. Magnification X 40. n = 6, ***P<0.001. (B) Immunofluorescent co-staining on the indicated pancreatic tissue for the epithelial marker cytokeratin19 (green), B7-1 (red) and DAPI nuclear counterstain (blue). Merge shows the co-localization of B7-1 with CK-19 (yellow) and is quantitated in the right panel. Magnification X 40. n = 3, ***P<0.001.

Several studies have reported that the immune checkpoint protein B7-1 is more highly expressed in human pancreatic tumors than normal pancreatic tissue [17, 18]. This was further documented in Fig 1 where we not only show increased B7-1 staining in pancreatic cancer tissue (Fig 1A), but that expression colocalizes with the epithelial marker CK19 (Fig 1B). CK-19 is mainly expressed in ductal epithelial (bile and pancreatic duct, renal collecting ducts) and gastrointestinal epithelia [31, 32]. In the pancreas, CK-19 expression is usually observed in the exocrine ducts, but not in the exocrine acinar and endocrine islet cells [31].

**Fig 2:**
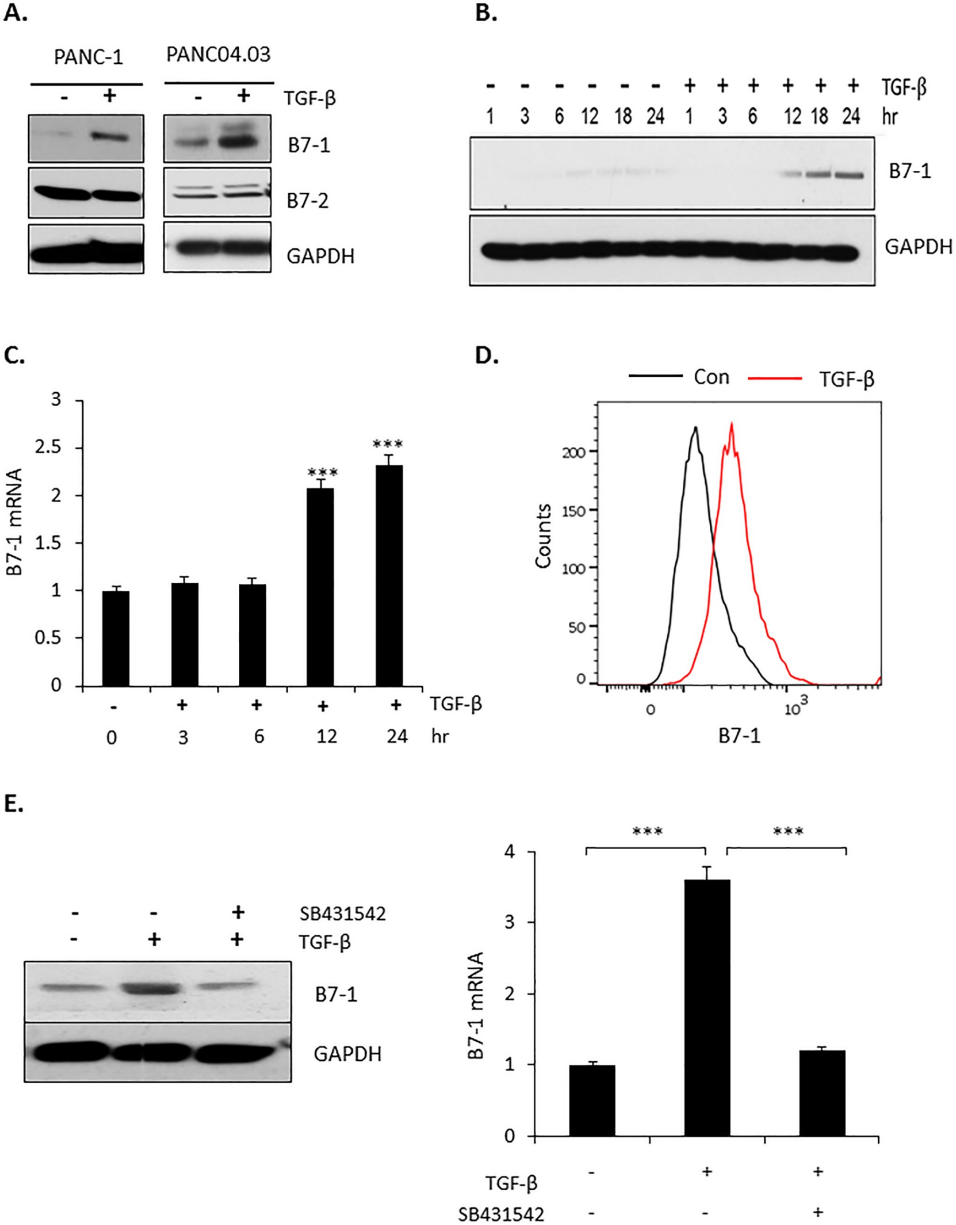
TGF-β increases expression of B7-1 (CD80) in human pancreatic cancer cells. (A) Pancreatic cancer cell lines PANC-1 or PANC04.03 were treated in the absence (-) or presence (+) of 5 ng/ml TGF-β for 24 hr and Western blotted for the indicated proteins as described in Materials and methods. (B) PANC04.03 cells were stimulated in the absence (-) or presence (+) of 5 ng/ml TGF-β and processed as in (A) for B7-1 or GAPDH at the indicated times. (C) PANC04.03 cells were treated as in (B) and total RNA (500 ng) subjected to RT-qPCR analysis for B7-1 (n = 3). ***P<0.001. (D) Cell surface expression of B7-1 was confirmed by flow cytometry in PANC04.03 cells treated as in (A). (E) PANC04.03 cells were untreated (-) or treated (+) with TGF-β (5 ng/ml) and/or the TβRI kinase inhibitor SB431542 (10 μM) for 24 hr prior to Western blotting (left panel) or RT-qPCR (500 ng; right panel) for B7-1 expression. ***P<0.001.

Tumor progression/metastasis reflects a highly regulated interaction between both intrinsic and extrinsic factors [33]. Since TGF-β has a critical role in numerous homeostatic as well as pathological processes [34, 35], we investigated the inter-relation of the growth-promoting/migratory role of TGF-β with the expression of B7-1 in pancreatic cancer cells. As shown in Fig 2A, TGF-β induced the expression of B7-1, but not B7-2, in PANC-1 and PANC04.03 cells. Kinetic analysis showed increased B7-1 protein and gene expression significantly increased by TGF-β beginning within 6–12 hr and maintained for at least 24 hr (Fig 2B and 2C). Since B7-1 is a surface glycoprotein [36], we confirmed cell surface B7-1 expression following exposure to TGF-β using FACS analysis (Fig 2D). Last, to further document that the observed increase in B7-1 protein and mRNA was specific to TGF-β receptor activation, treatment of PANC04.03 cells with the type I TGF-β receptor (TβRI) kinase inhibitor SB431542 was shown to abrogate the response (Fig 2E).

### Loss of B7-1 prevents TGF-β mediated pancreatic tumor cell migration and invasion

**Fig 3:**
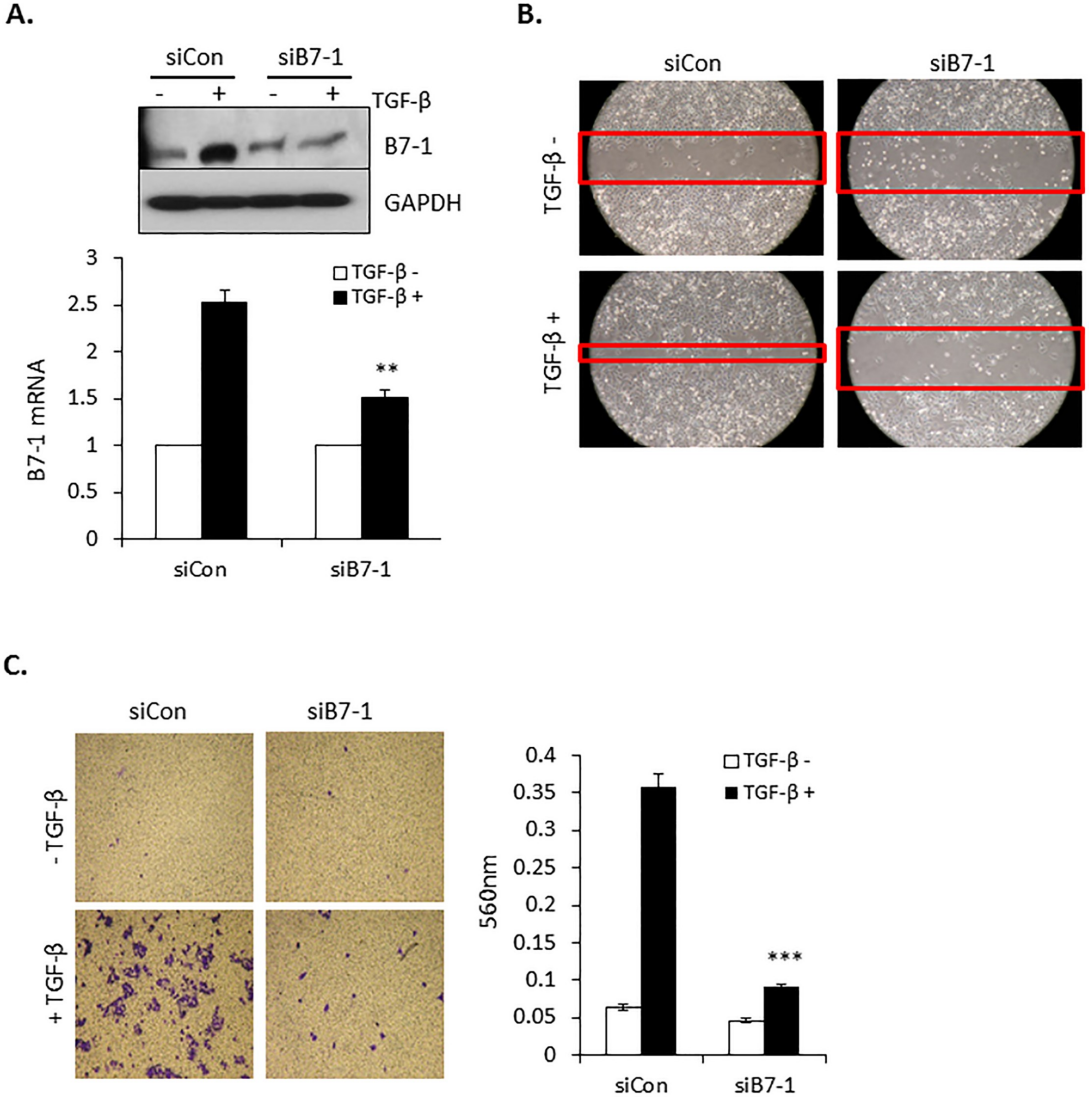
Knockdown of B7-1 inhibits PANC04-03 cell migration and invasion induced by TGF-β. (A) PANC04.03 cells were transiently transfected with either non-targeting (siCon) or siRNA to B7-1. Following 24 hr stimulation in the absence (-) or presence (+) of 5 ng/ml TGF-β cultures were Western blotted for B7-1 and GAPDH (top panel) or processed for B7-1 expression by RT-qPCR (bottom panel; **P<0.01 and n = 3). (B) Scratch assays were performed in control (siCon) and B7-1 knockdown PANC04.03 cells treated with (+) or without (-) TGF-β (5 ng/ml) for 24 hr and are representative of 3 separate experiments. Red bands indicate the leading edge. (C, left panels) Transwell invasion assays were performed in control and B7-1 knockdown PANC04.03 cells treated as in (B). (C, right panels) Quantitation of invasion. Data reflect mean ± SEM of n = 3, ***P<0.001.

Immune checkpoint proteins have been reported to provide tumors with both a growth and survival advantage [37, 38]. In that TGF-β is similarly known to promote tumor progression and invasion [39], we next investigated whether there was any causal relationship between the induction of B7-1 (Fig 2) and TGF-β’s protumorigenic activity. To address this question, siRNA was used to knockdown B7-1 protein/mRNA (Fig 3A). While the protein level of B7-1 in the absence of TGF-β was essentially the same in the siCon and siB7-1 cultures, this simply reflects the baseline value as the actual fold level of B7-1 mRNA following siB7-1 treatment was 0.47 relative to siCon. Most importantly, while B7-1 mRNA in siCon treated cells increased 2.7 fold with TGF-β, treatment with siB7-1 prevented any increase by TGF-β (i.e., went from 0.47 to 0.5 in siB7-1 cultures -/+ TGF-β, respectively). This was similarly seen in the Western analysis where siB7-1 abrogated the stimulatory effect of TGF-β on B7-1 protein expression. Following B7-1 knockdown, the cellular response to TGF-β stimulated cell migration (Fig 3B) and transwell invasion (Fig 3C) was determined. For both assays, the absence of B7-1 significantly inhibited TGF-β action.

### B7-1 regulates TGF-β induced EMT in PANC04.03 cells

**Fig 4:**
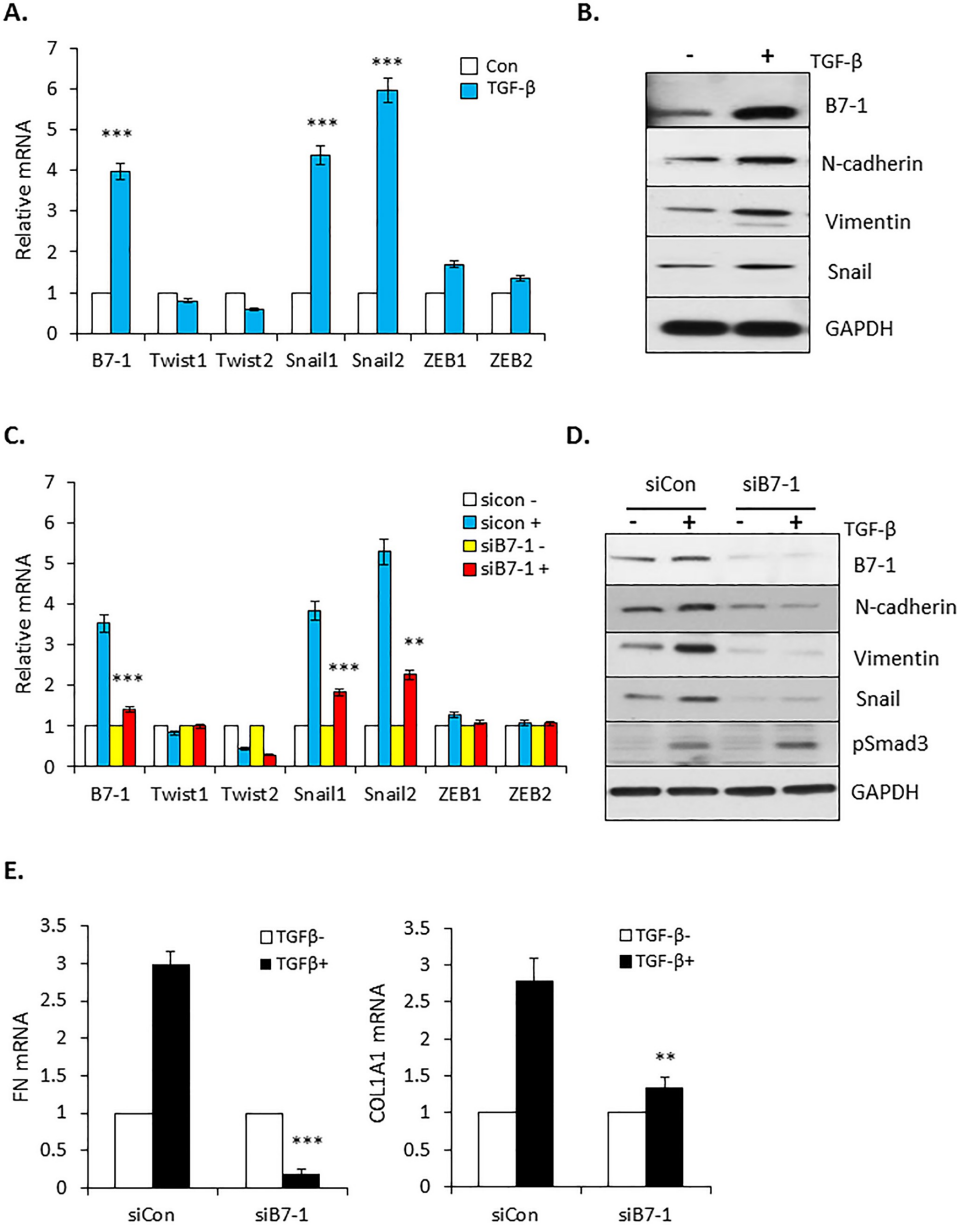
B7-1 is required for TGF-β-mediated EMT. (A) RT-qPCR expression of B7-1 and Snail, Twist, and ZEB following 24 hr stimulation of PANC04.03 cells in the absence (Con) or presence of 5 ng/ml TGF-β. ***P<0.001 and n = 3. (B) Cells were treated with (+) or without (-) TGF-β (5 ng/ml) for 24 hr and total cell lysates subjected to Western blot analysis for expression of the indicated proteins. (C) PANC04-03 cultures were transfected with non-targeting (siCon) or B7-1 siRNA as in Fig 3A and processed for RT-qPCR subsequent to 24 hr vehicle (-; 4 mM HCl, 0.1% BSA) or TGF-β (+; 5 ng/ml) treatment. **P<0.01, ***P<0.001 and n = 3. (D and E) siCon or siB7-1 PANC04.03 cells were stimulated ± TGF-β as in (C) and assessed by Western blotting (D) or RT-qPCR (E) for the indicated targets. **P<0.01, ***P<0.001 and n = 3 for E.

Primary *in vitro* evidence reflecting the pro-tumorigenic actions of TGF-β is the epithelial-to-mesenchymal transition (EMT) where the epithelial phenotype associated with cell-cell contact and apical-basal polarity is lost [40–42]. Since this process has been reported to be regulated by several transcription factors including paralogs of the Snail, Twist, and/or ZEB families [43–45], we assessed the effect of TGF-β on their expression in PANC04.03 cells by RT-qPCR. As shown in Fig 4A, while TGF-β treatment had no significant impact on the expression of Twist1/2 or ZEB1/2, Snail1 and Snail2 were induced 4–6 fold. Given that Snail transcription factors are known to regulate the expression of various mesenchymal markers associated with tumor invasion, metastasis, and cell motility [46–48], we determined, *first*, whether there was an analogous relation between the induction of Snail1/2 and profibrotic targets connected with TGF-β mediated EMT (Fig 4B); and most importantly, *second*, if B7-1 was required for the response (Fig 4C–4E). As shown in Fig 4B, coincident with the increase in Snail protein, treatment of PANC04.03 cells with TGF-β induced expression of the mesenchymal markers N-cadherin and Vimentin. Of particular note, not only was the increase in Snail mRNA and protein by TGF-β dependent upon B7-1 (Fig 4C and 4D), but mesenchymal targets associated with the mesenchymal transition were similarly diminished in the absence of B7-1 (Fig 4D and 4E). Thus, B7-1 has a fundamental role in promoting both the biologic and mechanistic aspects of the TGF-β driven EMT in PANC04.03 cells.

### B7-1 induction by TGF-β is dependent upon both Smad-dependent and -independent pathways

**Fig 5:**
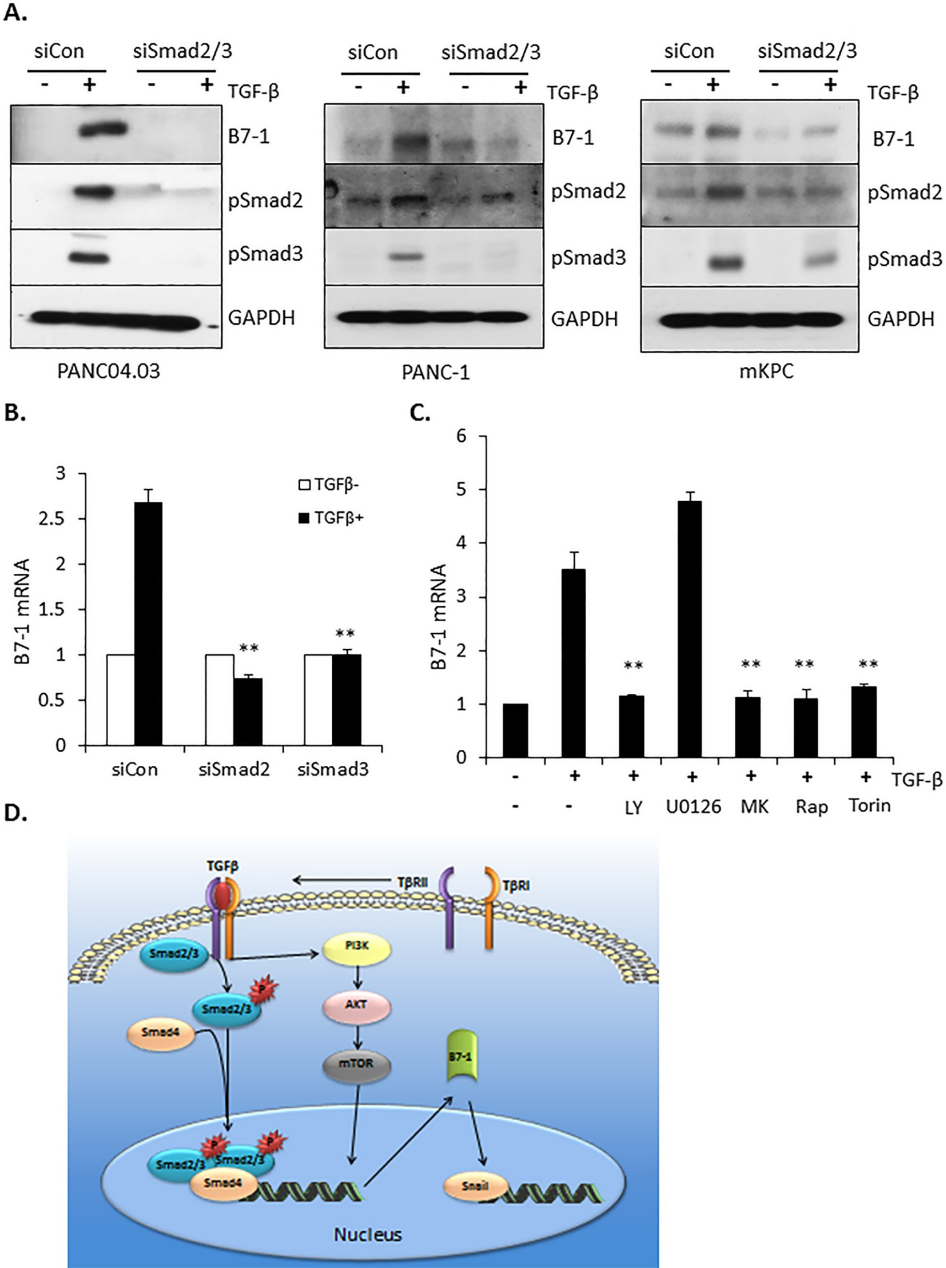
B7-1 induction by TGF-β is dependent on pSmad2/3 and PI3K pathways. (A) PANC04.03 cells were transfected with either non-targeting (siCon) or Smad2/3-targeting siRNA (60 nmol/L). After 72 hours of cultivation, the transient transfectants were stimulated in the absence (-) or presence (+) of 5 ng/ml TGFβ for 24 hr and Western blotted for B7-1, pSmad2, pSmad3, and GAPDH as a loading control. (B) Following transfection with the indicated siRNA and ± TGF-β treatment as in (A), RT-qPCR was performed for B7-1 gene expression at 24 hr. **P<0.01 and n = 3. (C) PANC04.03 cells were stimulated in the absence (-) or presence (+) of TGF-β (5 ng/ml) and the indicated compounds (10 μM LY294002 [LY], PI3K inhibitor; 10 μM U0126, MEK inhibitor; 300 nM MK2206 [MK], AKT inhibitor; 100 nM Rapamycin [Rap], mTORC1 inhibitor; or 200 nM Torin, mTORC1,2 inhibitor). Gene expression of B7-1 was determined as in (B). **P<0.01 and n = 3. (D) Model depicting the induction/role of B7-1 in TGF-β-mediated pancreatic cancer cell migration and induction of EMT target genes.

The preceding data show that B7-1 is highly expressed in pancreatic cancer (Fig 1), induced by TGF-β in two pancreatic cancer cell lines (Fig 2), and required for the induction of EMT in PANC04.03 cells (Figs 3 and 4). Since TGF-β signals via both Smad-dependent [49–51] as well as Smad-independent pathways (i.e., Erk, SAPK/JNK, mTOR, PI3K, and p38 MAPK) [27, 52, 53], we utilized both a genetic as well as pharmacologic approach to determine the operative pathway(s) regulating B7-1 induction. PANC04.03 cultures were first treated with siRNA targeting Smad2/3 or NT control and the effect on B7-1 protein and mRNA induction by TGF-β determined. As shown in Fig 5A and 5B, knockdown of Smad2 and/or Smad3 essentially abrogated the ability of TGF-β to induce B7-1. In that TGF-β is known to regulate target gene expression via multiple mechanisms [53–57], we assessed the role of the PI3K and MAPK pathways as they reflect the most upstream non-Smad activities coupled to the activated TGF-β receptor complex [27]. By using pharmacologic inhibitors, it was observed that while inhibition of the MAPK pathway with the MEK inhibitor U0126 had no negative impact on B7-1 expression by TGF-β, inhibition of PI3K and/or its downstream mediators Akt and mTOR abrogated the response (Fig 5C). A model depicting these findings is provided in Fig 5D.

## Discussion

Pancreatic cancer is the fourth leading cause of cancer-related deaths among both men and women with a 5 year survival rate in the USA of around 7–8% [58]. The high number of pancreatic cancer-related deaths is reflective of a number of elements including delayed diagnosis, natural resistance to chemotherapy or radiation therapy, and the relatively weak immune responses elicited by pancreatic antigens [59]. This latter finding is likely due to a combination of factors including the gain and/or loss of various oncogenes or tumor suppressor genes as well as the expression of checkpoint proteins such as B7-1 capable of dampening the immune response [60].

B7-1 is a transmembrane protein normally expressed on antigen presenting cells capable of providing either a co-stimulatory or co-inhibitory signal depending upon whether it interacts with its counter-receptor CD28 or CTLA4, respectively [61, 62]. The expression has been reported to portend a poor prognosis for patients with pancreatic cancer [63] and the combination of B7-1 and B7-H1 has been suggested as a diagnostic factor [18]. As such, determining the manner(s) by which B7-1 is regulated and its role in pancreatic cancer progression is critical to developing effective immunotherapeutic approaches for this deadly disease as recently shown for other solid tumors such as lung, melanoma, head and renal cancers [64–67]. To that end, as TGF-β is a known mediator of tumor progression/EMT and immune regulation [68], we addressed the following general questions: *first*, does TGF-β regulate B7-1 expression in pancreatic cancer cell lines; and if so, *second*, would B7-1 have a critical role in TGF-β stimulated cell migration/invasion and the induction of genes critical to EMT development?

In this study, we demonstrate that B7-1 protein is upregulated in human pancreatic cancer and TGF-β stimulates B7-1 expression in pancreatic cancer cell lines via both Smad-dependent as well as -independent pathways (Figs 1, 2 and 5). While B7-1 was induced by TGF-β in human PANC-1 and PANC-0403 as well as murine mKPC cells (Figs 2A and 5A), this was not observed in human BXPC-3 or MIA PaCa-2 cultures (not shown). This is the first time, to our knowledge, that B7-1 expression is described being regulated by TGF-β signaling and further document both the various responses of transformation to TGF-β and the importance of personalized approaches.

TGF-β is a known growth suppressor of pancreatic epithelial cells [20, 21]. While it is currently unknown if B7-1 has any role in this process, as TGF-β ligands are commonly overexpressed in pancreatic cancer and can promote epithelial to mesenchymal transition (EMT) and invasion [69, 70], we next investigated whether B7-1 was required for the induction of TGF-β target/regulatory genes and biological phenotypes associated with pancreatic cell EMT. To initially address that question the effect of B7-1 knockdown on TGF-β-stimulated cell migration and transwell invasion were assessed in PANC04.03 cells. Somewhat surprisingly, in the absence of B7-1 both responses were significantly inhibited (Fig 3). Since these activities are associated with the TGF-β regulated EMT, it was subsequently determined whether this was coincident with an inability to express EMT-inducing transcription factors such as Snail1/2, Twist1/2, and/or Zeb1/2 [71]. While Snail1/2 mRNA and protein were significantly increased by TGF-β, there was no appreciable impact on Twist1/2 or Zeb1/2 mRNA expression (Fig 4). Moreover, in the absence of B7-1 the TGF-β induction of Snail was diminished with a concomitant loss in EMT marker proteins (i.e., N-cadherin and Vimentin) as well as fibronectin and collagen α1 mRNA (Fig 4). As B7-1 is believed critical to the genesis and progression of tumor disorders, these findings (i) further document the various pathways and mechanisms by which TGF-β can regulate cell proliferation; and, most importantly, (ii) are consistent with the possibility that B7-1 inhibition might reflect a novel and effective means to impact both intrinsic and extrinsic aspects of pancreatic cancer.
